# Association between Gamma-Glutamyl Transferase and Coronary Atherosclerotic Plaque Vulnerability: An Optical Coherence Tomography Study

**DOI:** 10.1155/2019/9602783

**Published:** 2019-03-04

**Authors:** Jun Wang, Xing Li, Jun Pu, Siyu Jin, Lu Jia, Xiaomei Li, Fen Liu, Chunfang Shan, Yining Yang

**Affiliations:** ^1^Department of Coronary Heart Disease, the First Affiliated Hospital of Xinjiang Medical University, Urumqi 830011, China; ^2^The Renji Hospital of Shanghai Jiao Tong University, Shanghai 200240, China

## Abstract

**Background:**

Gamma-glutamyl transferase (GGT) has been detected in coronary plaques. However, the association between serum GGT levels and coronary atherosclerotic plaque vulnerability in patients with coronary artery disease (CAD) as detected by optical coherence tomography (OCT) has not been investigated.

**Methods:**

We performed a retrospective study of consecutively enrolled CAD patients undergoing preintervention OCT examination during coronary angiography. Plaque vulnerability was defined as the presence of ruptured plaques or thin-cap fibroatheroma (TCFA) upon OCT. The association between serum GGT levels and coronary plaque vulnerability was evaluated using multivariate logistic regression analysis.

**Results:**

A total of 142 patients were included in our analysis. OCT examination detected ruptured plaques in 16 patients, nonruptured plaques with TCFA in 17 patients, and nonruptured plaques and non-TCFA in 109 patients. Univariate analyses showed that gender, diabetes, Apolipoprotein A1 (ApoA1) and high-density lipoprotein cholesterol (HDL-c), and diagnosis of acute coronary syndrome (ACS) were associated with plaque vulnerability (P all < 0.05). Patients grouped according to serum GGT tertiles did not differ statistically in baseline characteristics or OCT findings. Results of multivariate logistic analyses showed that diabetes and diagnosis of ACS were associated with plaque rupture and TCFA (P < 0.05).

**Conclusions:**

GGT serum levels were not associated with OCT detected coronary vulnerability in our cohort of CAD patient.

## 1. Introduction

Pathologically, coronary lesion vulnerability is a key determinant of acute coronary syndrome (ACS) [1]. The potential pathophysiological mechanisms underlying vulnerable plaque rupture include hemodynamic changes, inflammation, oxidative stress, and conventional risk factors for coronary artery disease (CAD), including smoking, obesity, and diabetes [2, 3]. However, some healthy individuals who do not present with these risk factors can also develop ACS [2, 3]. Therefore, identification of new risk factors to explain individual variation in cardiovascular risk is very important.

Gamma-glutamyl transferase (GGT) is a common enzyme expressed on the cell membrane and distributed in the plasma [4]. GGT is a commonly used indicator of liver function because it is low-cost, highly sensitive, and accurate [5]. Elevated serum GGT levels have been found in patients with hepatobiliary disease and alcohol abuse [6]. Interestingly, some recent studies indicated a potential role of GGT in the diagnosis and prognosis of CAD [7, 8]. Moreover, pathological studies demonstrated that GGT was detected in coronary atherosclerotic plaques, suggesting that GGT is involved in CAD pathogenesis [9]. GGT mediates glutathione degradation and leads to the oxidation of low-density lipoprotein cholesterol (LDL-C), which accumulates in the artery wall and causes atherosclerosis [10]. Moreover, GGT located in the atherosclerotic plaque may increase lesion vulnerability by enhancing oxidative stress, cellular apoptosis, plaque rupture, and subsequent thrombosis [11]. Accordingly, previous studies suggested that GGT may serve as a risk marker of cardiovascular diseases, especially CAD [12, 13]. Indeed, higher GGT has been associated with CAD incidence [14, 15], which can stably persist over time [16]. Similarly, a large-scale cohort study including 163,944 subjects demonstrated that GGT was independently associated with cardiovascular mortality, CAD, congestive heart failure, and ischemic or hemorrhagic stroke during a 17-year follow-up period [17]. Also, each standard deviation increment in log-GGT is associated with a 24% increase in 3-year mortality in ACS patients after percutaneous coronary intervention (PCI) [18]. Furthermore, higher serum GGT levels have been associated with conventional CAD factors, including diabetes, hypertension, and metabolic syndrome [19]. However, evidence is lacking regarding the direct association between serum GGT levels and coronary plaque vulnerability* in vivo*.

Currently, optical coherence tomography (OCT) is the most reliable intraluminal imaging technique for coronary plaque detection and can be used to precisely evaluate coronary lesion vulnerability [20]. To the best of our knowledge, the potential association between serum GGT levels and OCT evidenced coronary vulnerability has not been reported. Therefore, in this study, we investigated if elevated serum GGT levels can predict incidence of plaque vulnerability, defined as plaque rupture or thin-cap fibroatheroma (TCFA) detected by OCT, in CAD patients.

## 2. Methods

### 2.1. Patient Selection

**Figure 1:**
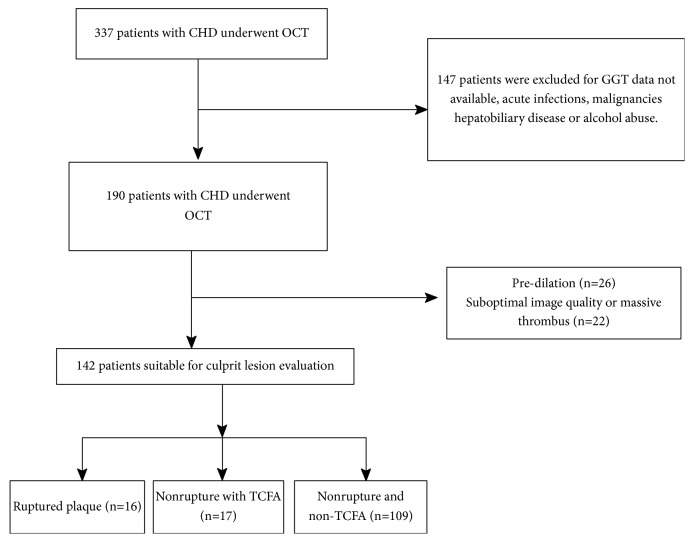
Flowchart of patient enrollment.

We conducted a single center retrospective cohort study at the First Affiliated Hospital of Xinjiang Medical University. Patients diagnosed with CAD who underwent preintervention OCT examination during coronary angiography (CAG) from January 2015 to October 2018 were included. Patients with the following clinical conditions were excluded: decreased white blood cell counts, decreased platelet counts, autoimmune disease, severe renal dysfunction (serum creatinine ≥ 265 *μ*mol/L or eGFR < 90 (ml/min/1.73m^2^)), history of hepatitis or positive detection of serum hepatitis B virus antigen, malignant tumors, history of alcohol abuse (defined as alcohol consumption ≥ 100 g/day), and basic liver disease including biliary obstructive disease, acute chronic viral hepatitis, drug-induced hepatitis, and fatty liver that affects GGT levels or alanine aminotransferase (the reference values of our laboratory for serum are 9–50 U/L) more than the triple normal upper limit. All participants provided written informed consent and the study protocol was approved by the ethics committee of the First Affiliated Hospital of Xinjiang Medical University. The flow chart of participant enrollment is shown in Figure 1.

### 2.2. Definitions of Cardiovascular Risk Factors

Demographic data, cardiovascular risk factors, and laboratory data were recorded for all patients. Systolic and diastolic blood pressure (SBP and DBP, respectively) were obtained as the average of two physician-obtained measurements using a mercury sphygmomanometer and taken after participants had rested for at least 5 minutes in a sitting position. Hypertension was defined if a patient was actively being treated with antihypertensive drugs or if blood pressure measurements were ≥ 140/90 mmHg on at least three separate occasions [21]. Diabetes mellitus was diagnosed according to the World Health Organization (WHO) criteria or if the patient was using hypoglycemic agents or insulin [22]. The diagnostic criteria for hyperlipidemia were in accordance with the 2016 Chinese Guideline for Prevention and Treatment of Dyslipidemia in Adult Patients [23]. Height and weight were recorded with the participants wearing light clothing and no shoes. Body mass index (BMI) was calculated by dividing the body weight (kg) by the height (m^2^). A BMI > 28 kg/m^2^ was considered obese [24]. Current smoking was self-reported and was defined as regular cigarette smoking within the prior year. Left ventricular ejection fraction (LVEF) was evaluated by echocardiography within 24 to 48 hours before CAG. We assessed the estimated glomerular filtration rate (eGFR) according to the Modification of Diet in Renal Diseases equation [25]. Alcohol consumption was defined as alcohol consumption 50 g/day.

### 2.3. Measurement of Serum GGT

Blood samples were immediately centrifuged, and plasma and serum specimens were stored at −20°C until assayed. Serum GGT activity was measured using spectrophotometry at 405 nm, which detects the liberation of* p*-nitroaniline, resulting from the reaction of gamma-glutamyl-*p*-nitroanilide + glycylglycine (Quest Diagnostics [MedPath]) [26].

### 2.4. CAG and OCT Analyses

**Figure 2:**
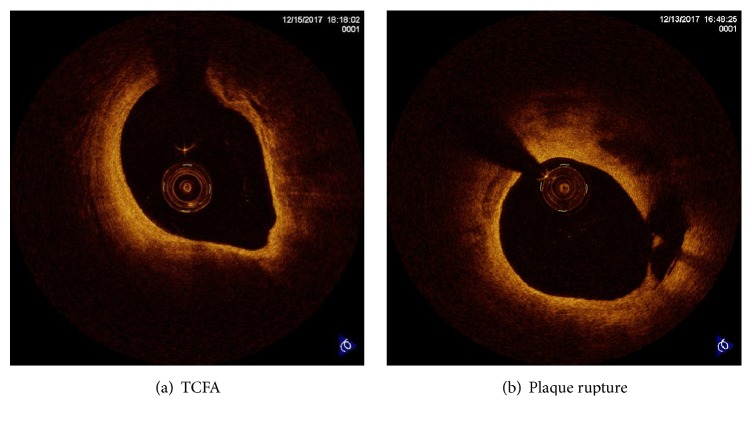
Representative OCT images of TCFA (a) and plaque rupture (b).

CAG was performed using a standard procedure by experienced interventional cardiologists. We used a commercially available C7-XR OCT intravascular imaging system (OCT C7 Dragonfly, St. Jude Medical, St Paul, MN, USA) for OCT analyses. All target lesions before balloon dilatation were examined using standard OCT. The proximal end of the OCT catheter was threaded to the distal end of the lesion, together with the root. The above steps were repeated according to the length of the target vessel and imaging quality, and the positioning of branches or calcification was selected as far as possible. Two or three retractions were performed to complete the target vessel examination, and the distance from the lesion to the opening was determined by two experienced interventionists. Plaque lipid content was semiquantitatively evaluated (angle or quadrant). The thinnest part of the fibrous cap covered by the lipid pool was measured three times, with the average value recorded. Two independent observers performed offline analysis of OCT images in accordance with the established OCT diagnostic criteria to eliminate poor quality images. TCFA was defined as a plaque with a maximal lipid arc > 90° and thinnest fibrous cap thickness ≤ 65 *μ*m (Figure 2(a)) [27, 28]. Plaque rupture was identified by fibrous cap discontinuity with a cavity formed inside the plaque (Figure 2(b)) [27, 28].

### 2.5. Statistical Analysis

All analyses were performed using SPSS 24.0 for Windows statistical software (SPSSInc, Chicago, IL, USA). The sample size of the study was estimated by the following formula: $n=2\overset{-}{p\;}\overset{-}{q}{\left(u_{a}+u_{\beta }\right)}^{2}/{\left(p_{1}-p_{0}\right)}^{2}$. Continuous variables are expressed as mean ± standard deviation or median (25th to 75th percentiles) values, and categorical variables are presented as percentages. The differences between normally distributed numeric variables were evaluated using one-way ANOVA, and nonnormally distributed variables were analyzed using the Mann–Whitney* U* test or Kruskal–Wallis variance analysis as appropriate. The Chi-square (*χ*2) test was used to compare categorical variables. We classified patients into three groups according to GGT tertiles in the subsequent analyses. To construct the model for multivariate regression analyses, univariate models for each of the predictor variables were run, and the variables that were significant (P < 0.05) in univariate analysis were entered into multiple linear regression and logistic regression analyses. The odds ratios (OR) and 95% confidence intervals (CIs) were calculated. P < 0.05 was considered significant.

## 3. Results

### 3.1. Characteristics of Patients according to Plaque Vulnerability

A total of 142 patients with CAD were included in this study. The baseline characteristics of coronary risk factors and biochemical parameters according to plaque vulnerability as detected by OCT are presented in Table 1. Gender distribution, prevalence of diabetes, levels of Apolipoprotein A1 (ApoA1) and high-density lipoprotein cholesterol (HDL-c), and the proportions of patients with ACS diagnoses were significantly different among the groups. Patients with ruptured plaque or nonrupture with TCFA were more likely to be male, diabetic, dyslipidemic, and diagnosed with ACS compared to those with nonrupture and non-TCFA (P all < 0.05). GGT plasma levels were not statistically different among the three groups.

### 3.2. CAG Findings and OCT Analysis

CAG findings and OCT analysis are shown in Table 2. Although the primary CAG findings were not significantly different among the three groups, OCT analysis showed considerable differences in minimal fibrous cap thickness, lipid arc, macrophage accumulation, TCFA, plaque characteristics, and thrombus formation among the three groups (P all < 0.05).

### 3.3. Association between Patient Characteristics and Coronary Vulnerability Determined by OCT

Model 1 indicates the outcome of the plaque rupture group versus the nonplaque rupture with TCFA group, and model 2 indicates the outcome of the nonplaque rupture with TCFA group versus the nonrupture and non-TCFA group. Results of multivariate logistic analyses showed that diabetes (OR: 5.879, P = 0.006) and ACS (OR: 6.876, P = 0.009) were independently associated with plaque rupture and presence of TCFA as determined by OCT (Table 3).

### 3.4. Relationship of GGT with Patient Characteristics and OCT Findings

Patients were divided into three groups according to tertiles of GGT activity: 1st tertile (GGT < 23 U/L; n = 48), 2nd tertile (GGT ≥ 23 U/L to 38 U/L; n=47), and 3rd tertile (GGT > 38 U/L; n = 47). Baseline data in patients with different GGT levels are shown in Table 4. Age, male, oral antidiabetic agents, insulin use, current smoking, current drinking, obesity, levels of triglycerides (TG), alanine aminotransferase (ALT), carbamide, eGFR, unconjugated bilirubin (IBiL), and *β*-blockers were significantly different across the GGT tertiles (all P < 0.05). Incident of proximal target plaque in the 1st tertile was higher compared to the 2nt tertile combined with the 3rd tertile (P = 0.014). Moreover, OCT findings in patients among the three groups with different levels of GGT were not significantly different (Table 5).

## 4. Discussion

In this study, we found that circulating GGT levels were not associated with an increased risk of coronary lesion vulnerability, as indexed by TCFA and plaque rupture using OCT analysis, in patients with CAD. The pathophysiological mechanisms underlying the potential association between GGT and CAD incidence and prognosis may not have previously included contribution of GGT to plaque vulnerability.

Previous studies reported an association between GGT and CAD risk and prognosis. A prospective study including 469 patients with ischemic syndrome and CAG documented CAD confirmed that GGT activity is an independent prognostic marker of incidence of cardiac death and infarction [29]. In addition, a positive and independent correlation between baseline levels of GGT and risk of sudden cardiac death in the general male population was confirmed in a cohort study with a 22-year follow-up [30]. Moreover, high serum GGT levels have been independently and significantly correlated with coronary artery calcification score progression in an asymptomatic middle-aged population [31]. However, these studies focused on the association between GGT and CAD incidence and prognosis, rather than the potential association between GGT and coronary lesion characteristics, which previously limited our understanding of the insights into the potential association between GGT and coronary atherosclerotic plaque vulnerability. Our study used OCT, referred to as a histologic microscope* in vivo* [32], and showed that higher GGT levels were not associated with plaque vulnerability. Interestingly, several studies revealed that elevated GGT in CAD patients may be explained by alcohol consumption but that light to moderate alcohol drinking may have a protective effect against CAD and myocardial infarction [33–35]. However, these hypotheses are challenged by recent findings that do not support a protective effect of alcohol consumption [36, 37]. Therefore, the potential mechanisms underlying the association between GGT and CAD require further investigation. However, previous studies have provided some insight into the potential mechanisms underlying the association between GGT and CAD. GGT is the hydrolytic enzyme of extracellular glutathione (GSH), a main antioxidant factor* in vivo *[4]. By hydrolyzing GSH, GGT causes an imbalance of LDL oxidation and leads to overproduction of oxidized LDL (ox-LDL), a key component of atherosclerotic plaques [38]. Therefore, GGT may directly participate in the development of atherosclerosis by mediating the oxidative stress response [39, 40]. Thus, GGT may be involved in the pathogenesis of atherosclerotic plaque formation, but not plaque vulnerability.

Numerous clinical epidemiological studies have suggested that elevated GGT levels are not only related to liver diseases and alcohol consumption but also closely related to the incidence and development of many systemic diseases, such as hypertension, serum hyperlipemia, diabetes, and metabolic syndrome [4, 17, 41, 42]. GGT might be a potentially reliable, simple, and noninvasive biochemical marker for determining cardiovascular risk, which may be helpful for successful disease prognosis. However, further studies including larger populations are necessary to confirm this conclusion. Consistent with previous studies, our univariate analysis showed that age, male, oral antidiabetic agents, insulin use, current smoking, current drinking, obesity, and increased TG were significantly different when considering GGT levels. These results also confirmed that circulating GGT may be closely correlated with conventional risk factors for CAD incidence, rather than plaque vulnerability.

### 4.1. Study Limitations

Our study has several limitations. First, depth of OCT does not permit us to assess plaque volume or positive remodeling of atheromatous plaques. Secondly, we only analyzed plaque composition at the site of target lesions; thus, the association between diabetes, obesity, and coronary vulnerability in nontarget lesions should also be determined in future studies. Thirdly, we only collected baseline GGT data during the study duration. Therefore, the effect of dynamic changes of GGT on plaque vulnerability could not be determined. Fourth, no inflammatory markers, such as high-sensitive C reactive protein and ox-LDL, were assessed in this study, and these factors may confound the results. Finally, this was a single center retrospective study, and our results need to be further verified with a multicenter, prospective study.

## 5. Conclusions

Serum GGT was not associated with coronary vulnerability as determined by OCT in our cohort. The pathophysiological mechanisms underlying the potential association between GGT and CAD incidence and prognosis as observed in previous clinical studies may not include contribution of* circulating GGT* levels to plaque vulnerability.

## Figures and Tables

**Table 1:** Characteristics of patients according to plaque vulnerability.

	Ruptured plaque	Nonrupture with TCFA	Nonrupture and non-TCFA	t/Z/*χ*^2^	P
Sex (Male/Female)	15/1	15/2	74/35	8.331	0.016
Age	59.63±10.15	54.65±9.23	56.89±11.99	0.773	0.463
Hypertension	10 (62.5)	9 (52.9)	57 (52.3)	0.587	0.746
Diabetes mellitus	10 (62.5)	10 (58.8)	25 (22.9)	16.657	<0.001
DM treatment					
Oral hypoglycemic drugs	6 (37.5)	2 (11.8)	16 (14.7)	4.614	0.100
Insulin	3 (18.8)	0 (0.0)	13 (11.9)	4.876	0.087
Diet only	0 (0.0)	0 (0.0)	4 (3.7)	2.168	0.338
Current smoking	9 (56.3)	9 (52.9)	60 (55.0)	0.039	0.981
Alcohol drinking	4 (25.0)	2 (11.8)	19 (17.4)	0.993	0.609
Family history of CAD	2 (12.5)	1 (5.9)	24 (22.0)	3.566	0.168
Previous myocardial infarction	1 (6.3)	3 (17.6)	21 (19.3)	2.004	0.367
Previous PCI	2 (12.5)	2 (11.8)	31 (28.4)	4.063	0.131
SBP (mmHg)	129.38±26.20	125.65±17.16	124.84±17.87	0.403	0.669
DBP (mmHg)	74.25±23.31	73.29±8.04	76.03±12.12	0.383	0.683
Obesity (BMI≥28kg/m^2^)	7 (43.8)	6 (35.3)	35 (32.1)	0.864	0.649
HDL-C (mmol/l)	0.83±0.15	0.89±0.09	1.07±0.30	7.380	0.001
LDL-C (mmol/l)	2.39±0.87	2.60±0.69	2.30±0.90	0.824	0.441
TC (mmol/l)	3.61±0.98	4.09±0.72	3.67±1.08	1.251	0.290
TG (mmol/l)	1.94±0.84	1.90±0.77	1.82±0.96	0.136	0.873
ApoA1 (g/L)	0.96±0.13	1.01±0.08	1.15±0.25	7.171	0.001
ApoB (g/L)	0.78±0.28	0.87±0.21	0.8±0.54	0.165	0.848
Lp (a) (g/L)	219 (147,358)	147 (57,326)	182 (96,386)	2.691	0.260
HbA1c (%)	7.07±1.34	5.96±0.36	6.36±1.33	1.586	0.212
ALT (U/L)	35.64±21.54	31.86±21.61	31.89±24.33	0.178	0.837
AST (U/L)	25.31±12.09	20.07±6.34	30.46±36.99	0.825	0.440
Creatinine (*μ*mmol/L)	77.54±17.24	74.3±16.51	74.15±19.72	0.221	0.802
BUN (mmol/l)	5.96±1.85	5.01±1.52	5.54±1.6	1.430	0.243
eGFR (ml/min/1.73m^2^)	110.28±47.59	107.07±29.18	106.76±37.72	0.060	0.942
Uric acid (*μ*mol/L)	356.47±72.48	338.83±79.78	331.33±99.4	0.506	0.604
TBil (mmol/l)	12.1±3.95	13.88±4.76	13.46±10.47	0.174	0.840
DBiL (mmol/l)	3.04±1.38	3.57±1.41	3.71±2.78	0.493	0.612
IBiL (mmol/l)	9.16±3.93	10.31±4.63	9.38±5.91	0.231	0.794
EF (%)	61.34±7.02	60.38±10.15	61.25±6.85	0.097	0.908
GGT (U/L)	25 (18,40)	32, (19,65)	28 (20,43)	1.708	0.426
GGT tertiles				2.843	0.584
1st tertile	6 (37.5)	6 (35.3)	35 (32.1)		
2st tertile	6 (37.5)	3 (17.6)	38 (34.9)		
3st tertile	4 (25.0)	8 (47.1)	36 (33.0)		
ALP	74.74±20.43	79.45±24.98	78.07±23.09	0.192	0.826
ACS	13 (81.3)	13 (76.5)	47 (43.1)	12.977	0.002
Aspirin	11 (68.8)	14 (82.4)	85 (78.0)	0.907	0.635
Statins	11 (68.8)	12 (70.6)	82 (75.2)	0.407	0.816
*β*-Blockers	7 (43.8)	3 (17.6)	43 (39.4)	3.307	0.191
ACEI/ARB	6 (37.5)	6 (35.3)	41 (37.6)	0.034	0.983
CCB	5 (31.3)	5 (29.4)	27 (24.8)	0.407	0.816
GRACE risk score	102.33±17.76	108.11±26.35	108.36±28.64	0.190	0.828

Data are presented as n (%) or mean ± SD.

BMI, body mass index; TG, triglyceride; TC, total cholesterol; HDL-C, high-density lipoprotein cholesterol; LDL-C, low-density lipoprotein cholesterol; TBil, total bilirubin; DBiL, direct bilirubin; IBiL, unconjugated bilirubin; Apo A1, Apo lipoprotein AI; Apo B, Apo lipoprotein B; Lp (a), lipoprotein (a); SBP, systolic blood pressure; DBP, diastolic blood pressure; ALT, alanine aminotransferase; AST, aspartate aminotransferase; ALP, alkaline phosphatase.

**Table 2:** CAG findings and OCT characteristics according to plaque vulnerability.

		Ruptured plaque	Non-rupture with TCFA	Non-rupture and non-TCFA	t/*χ*^2^	P
Erosion (%)	No	14 (87.5)	12 (70.6)	97 (89.0)	3.533	0.171
	Yes	2 (12.5)	5 (29.4)	12 (11.0)		
Macrophage accumulation	0	3 (18.8)	4 (23.5)	69 (63.3)	28.094	<0.001
	1	6 (43.8)	7 (41.2)	25 (22.9)		
	2	5 (37.5)	5 (29.4)	15 (13.8)		
	3	1 (6.3)	1 (5.9)	0 (0.0)		
	4	1 (6.3)	0 (0.0)	0 (0.0)		
Vasa vasorum	No	15 (93.8)	14 (82.4)	102 (93.6)	2.098	0.350
	Yes	1 (6.3)	3 (17.6)	7 (6.4)		
Thrombus	No	4 (25.0)	10 (58.8)	97 (89.0)	32.340	<0.001
	Yes	12 (75.0)	7 (41.2)	12 (11.0)		
Calcified nodule	No	16 (100.0)	17 (100.0)	101 (92.7)	4.374	0.112
	Yes	0 (0.0)	0 (0.0)	8 (7.3)		
Characteristic of plaque	Lipid	15 (93.7)	17 (100.0)	64 (58.7)	25.106	<0.001
	Calcified	1 (6.3)	0 (0.0)	21 (19.3)		
	Fibrotic	0 (0.0)	0 (0.0)	24 (22.0)		
Minimal lumen area (mm^2^)		3.46±1.89	3.72±1.95	3.34±1.88	0.258	0.773
Normal lumen area (mm^2^)		13.04±2.77	11.03±2.75	10.08±3.13	5.245	0.007
Diameter stenosis, %		83.69±12.23	73.24±14.36	76.85±12.76	2.867	0.060
Lesion length		9.54±4.18	9.73±2.94	10.22±3.5	0.355	0.702
Target vessel	LAD, n (%)	10 (62.5)	12 (70.6)	85 (78.0)	4.125	0.389
	LCX, n (%)	2 (12.5)	3 (17.6)	6 (5.5)		
	RCA, n (%)	4 (25.0)	2 (11.8)	18 (16.5)		
Location of target plaque	Proximal	10 (62.5)	12 (70.6)	72 (66.1)	0.245	0.885
	Mid-Distal	6 (37.5)	5 (29.4)	37 (33.9)		
TIMI classification	0	1 (6.3)	1 (5.9)	1 (0.9)	9.933	0.077
	1	0 (0.0)	0 (0.0)	2 (1.8)		
	2	2 (12.5)	3 (17.6)	5 (4.6)		
	3	13 (81.3)	13 (76.5)	101 (92.7)		
Number of vascular lesions	1	8 (50.0)	8 (47.1)	52 (47.7)	0.833	0.934
	2	5 (31.3)	4 (23.5)	34 (31.2)		
	3	3 (18.8)	5 (29.4)	23 (21.1)		

**Table 3:** Predictors of plaque vulnerability as detected by ruptured plaque or non-rupture with TCFA: multivariate logistic regression analysis.

	Model 1			Model 2		
	P	OR	95% CI	P	OR	95% CI
HDL-c	0.210	0.064	0.001-4.672	0.569	0.347	0.009-13.162
ApoA1	0.403	0.136	0.001-14.645	0.438	0.182	0.002-13.502
Diabetes	0.006	5.879	1.651-20.939	0.005	5.395	1.657-17.567
ACS	0.009	6.876	1.620-29.189	0.013	5.115	1.419-18.431
Sex	0.075	7.605	0.818-70.682	0.119	3.756	0.711-19.849

OR, odds ratio; CI, confidence interval.

**Table 4:** Characteristics of participants according to serum GGT tertiles.

	1st tertile	2nd tertile	3rd tertile	t/Z/*χ*^2^	P
Sex (Male/Female)	24/23	36/11	44/4	20.379	<0.001
Age	60.26±10.69	58.15±10.29	52.48±12.17	6.265	0.002
Hypertension	20 (42.6)	29 (61.7)	27 (56.3)	3.681	0.159
Diabetes mellitus	13 (27.7)	15 (31.9)	17 (35.4)	0.662	0.718
DM control					
Oral hypoglycemic agents	4 (8.5)	13 (27.7)	7 (14.6)	6.413	0.041
Insulin	4 (8.5)	10 (21.3)	2 (4.2)	7.488	0.024
Diet only	0 (0.0)	1 (2.2)	3 (6.3)	4.306	0.116
Current smoking	15 (31.9)	27 (57.4)	36 (75.0)	17.986	<0.001
Alcohol drinking	4 (8.5)	6 (12.8)	15 (31.3)	9.599	0.008
Family history of CAD	7 (14.9)	8 (17.0)	12 (25.0)	1.756	0.416
Previous myocardial infarction	12 (25.5)	4 (8.5)	9 (18.8)	4.759	0.093
Previous PCI	14 (29.8)	10 (21.3)	11 (22.9)	1.033	0.596
SBP (mmHg)	124.36±20.59	127.85±18.80	124.17±17.03	0.571	0.566
DBP (mmHg)	74.13±12.88	76.45±12.82	75.92±14.49	0.386	0.681
Obesity (BMI≥28kg/m^2^)	9 (19.1)	17 (36.2)	22 (45.8)	7.733	0.021
HDL-c (mmol/l)	1.03±0.26	1.07±0.32	0.96±0.27	1.788	0.171
LDL-c (mmol/l)	2.26±0.74	2.36±0.82	2.42±1.04	0.381	0.684
TC (mmol/l)	3.55±0.91	3.74±1.05	3.85±1.15	0.957	0.387
TG (mmol/l)	1.55±0.77	1.79±0.83	2.20±1.04	6.564	0.002
ApoA1 (g/L)	1.12±0.23	1.14±0.24	1.08±0.23	0.712	0.493
ApoB (g/L)	0.76±0.22	0.86±0.76	0.8±0.31	0.467	0.628
Lp (a) (g/L)	205 (76,353)	174 (97,404)	184 (119,347)	0.522	0.770
HbA1c (%)	6.03±1.05	6.67±1.52	6.48±1.21	1.443	0.243
ALT	22.52±12.5	30.56±24.57	43.59±26.43	11.021	<0.001
AST	23.66±14.85	30.1±43.46	32.07±33.76	0.846	0.431
Creatinine	73.84±21.96	74.71±17.96	75.09±17.22	0.054	0.948
BUN	5.25±1.49	6.01±1.65	5.3±1.65	3.329	0.039
eGFR	95.15±28.33	108.39±39.75	117.81±41.14	4.519	0.013
Uric Acid (*μ*mol/L)	315.75±91.44	343.14±92.4	346.07±98.19	1.492	0.228
TBil (mmol/l)	12.05±4.91	12.08±4.53	15.88±14.55	2.677	0.072
DBiL (mmol/l)	3.75±2.13	3.13±1.52	3.96±3.47	1.411	0.247
IBiL (mmol/l)	8.31±3.99	8.98±3.94	11.09±7.6	3.346	0.038
EF (%)	61.35±6.81	61.8±4.43	60.37±9.54	0.455	0.635
ALP	71.85±16.37	78.40±26.66	83.22±23.50	3.027	0.052
ACS	26 (55.3)	25 (53.2)	22 (45.8)	0.945	0.624
Aspirin	33 (70.2)	36 (76.6)	41 (85.4)	3.175	0.204
Statins	30 (63.8)	35 (74.5)	40 (83.3)	4.698	0.095
*β*-Blockers	12 (25.5)	17 (36.2)	24 (50.0)	6.117	0.047
ACEI/ARB	14 (29.8)	16 (34.0)	23 (47.9)	3.660	0.160
CCB	12 (25.5)	14 (29.8)	11 (22.9)	0.592	0.744
GRACE risk score	103.40±32.72	112.56±20.21	105.65±28.69	0.757	0.473

Abbreviations are the same as those in Table 1.

**Table 5:** CAG findings and OCT analysis in patients according to the three groups.

	Group	1st tertile	2nd tertile	3rd tertile	t/Z/*χ*^2^	P
FCT (*μ*m)		100 (40,200)	140 (60,200)	140 (60,220)	2.877	0.237
Lipid arc, degree		100 (0,165)	114 (0,180)	147 (15,202)	3.321	0.190
Rupture (%)	No	41 (87.2)	41 (87.2)	44 (91.7)	0.624	0.732
	Yes	6 (12.8)	6 (12.8)	4 (8.3)		
Erosion (%)	No	40 (85.1)	40 (85.1)	43 (89.6)	0.549	0.760
	Yes	7 (14.9)	7 (14.9)	5 (10.4)		
Macrophage accumulation	0	28 (59.6)	24 (51.1)	24 (50.0)	4.465	0.910
	1	10 (21.3)	14 (29.8)	14 (29.2)		
	2	8 (17.0)	8 (17.0)	9 (18.8)		
	3	1 (2.1)	1 (2.1)	0 (0.0)		
	4	0 (0.0)	1 (2.1)	1 (2.1)		
Vasa vasorum	No	44 (93.6)	45 (95.7)	42 (87.5)	2.374	0.305
	Yes	3 (6.4)	2 (4.3)	6 (12.5)		
Thrombus	No	37 (78.7)	37 (78.7)	37 (77.1)	0.050	0.975
	Yes	10 (21.3)	10 (21.3)	11 (22.9)		
Calcified nodule	No	45 (95.7)	43 (91.5)	46 (95.8)	1.033	0.597
	Yes	2 (4.3)	4 (8.5)	2 (4.2)		
Characteristic of plaque	Lipid	30 (63.8)	30 (63.8)	36 (75.0)	4.863	0.302
	Calcified	10 (21.3)	9 (19.1)	3 (6.3)		
	Fibrotic	7 (14.9)	8 (17.0)	9 (18.8)		
TCFA	No	36 (76.6)	39 (83.0)	37 (77.1)	0.714	0.700
	Yes	11 (23.4)	8 (17.0)	11 (22.9)		
Minimal lumen area (mm^2^)		3.79±2.42	3.41±1.66	3.09±1.54	1.312	0.273
Normal lumen area (mm^2^)		10.9±3.74	10.91±2.94	9.8±2.79	1.688	0.189
Diameter stenosis, %		75.53±11.9	79.15±13.41	76.9±13.82	0.918	0.402
Lesion Length		9.84±3.72	10.35±3.53	10.05±3.32	0.255	0.775
Target vessel	LAD, n (%)	39 (83.0)	34 (72.3)	34 (70.8)	5.400	0.249
	LCX, n (%)	4 (8.5)	2 (4.3)	5 (10.4)		
	RCA, n (%)	4 (8.5)	11 (23.4)	9 (18.8)		
Location of target plaque	Proximal	35 (74.5)	35 (74.5)	24 (50.0)	8.501	0.014
	Mid-Distal	12 (25.5)	12 (25.5)	24 (50.0)		
TIMI classification	0	0 (0.0)	2 (4.3)	1 (2.1)	4.433	0.673
	1	1 (2.1)	0 (0.0)	1 (2.1)		
	2	5 (10.6)	2 (4.3)	3 (6.3)		
	3	41 (87.2)	43 (91.5)	43 (89.6)		
Number of vascular lesions	1	21 (44.7)	20 (42.6)	27 (56.3)	3.722	0.445
	2	13 (27.7)	18 (38.3)	12 (25.0)		
	3	13 (27.7)	9 (19.1)	9 (18.8)		

Abbreviations are the same as those in Table 1.

## Data Availability

The data that support the findings of this study are available from the First Affiliated Hospital of Xinjiang Medical University but restrictions apply to the availability of these data, which were used under license for the current study and so are not publicly available. Data are however available from the authors upon reasonable request and with permission of the First Affiliated Hospital of Xinjiang Medical University.
